# Cardiovascular Risk Factors in Socioeconomically Disadvantaged Populations in a Suburb of the Largest City in Western Romania

**DOI:** 10.3390/biomedicines12091989

**Published:** 2024-09-02

**Authors:** Andreea Dumitrescu, Gabriela Mut Vitcu, Svetlana Stoica, Septimiu Radu Susa, Emil Robert Stoicescu

**Affiliations:** 1 Neoclinic Concept SRL, Calea Dorobantilor 3, 300134 Timisoara, Romania; andreea.dumitrescu@e-uvt.ro (A.D.); gabriela.mut@cardioprevent.org (G.M.V.); 2 Department of Kinetotherapy, Faculty of Physical Education and Sport, West Univestity of Timisoara, 300223 Timisoara, Romania; 3 Cardiology University Clinic, Department VI, “Victor Babes” University of Medicine and Pharmacy Timisoara, Eftimie Murgu Square No. 2, 300041 Timisoara, Romania; 4 Research Centre of Timisoara Institute of Cardiovascular Diseases, “Victor Babes” University of Medicine and Pharmacy Timisoara, Eftimie Murgu Square No. 2, 300041 Timisoara, Romania; 5 Doctoral School, “Victor Babes” University of Medicine and Pharmacy Timisoara, Eftimie Murgu Square 2, 300041 Timisoara, Romania; septimiu.susa@umft.ro; 6 Radiology and Medical Imaging University Clinic, ‘Victor Babes’ University of Medicine and Pharmacy Timisoara, Eftimie Murgu Square No. 2, 300041 Timisoara, Romania; stoicescu.emil@umft.ro; 7 Research Center for Pharmaco-Toxicological Evaluations, ‘Victor Babes’ University of Medicine and Pharmacy Timisoara, Eftimie Murgu Square No. 2, 300041 Timisoara, Romania; 8 Field of Applied Engineering Sciences, Specialization Statistical Methods and Techniques in Health and Clinical Research, Faculty of Mechanics, ‘Politehnica’ University Timisoara, Mihai Viteazul Boulevard No. 1, 300222 Timisoara, Romania

**Keywords:** cardiovascular disease, socioeconomic disparities, socioeconomically disadvantaged populations, cardiovascular risk factors, Western Romania, hypertension, diabetes, dyslipidemia, obesity, public health interventions

## Abstract

*Background and Objectives*: Cardiovascular disease (CVD) remains a major public health issue worldwide, disproportionately affecting socioeconomically disadvantaged populations due to the social determinants of health (SDOHs). In Western Romania, these populations are particularly vulnerable to CVD. This study aims to investigate the prevalence and impact of cardiovascular risk factors (CVRFs) among socioeconomically disadvantaged individuals in Western Romania and identify the primary CVRFs contributing to the high incidence of CVD within this population. *Materials and Methods*: A retrospective observational design was employed, utilizing data from the medical records of 1433 eligible individuals. The inclusion criteria were based on Eurostat’s EU-SILC benchmarks, focusing on severe material deprivation, at-risk-of-poverty rates, and low work intensity. Data on demographics, familial and personal medical history, smoking status, blood pressure, glucose, cholesterol, triglycerides, and HbA1c levels were collected. *Results*: Of the 1433 subjects, 34.75% were male, with a median age of 52 years. Significant conditions included diabetes (7.39%), coronary disease (3.83%), arterial hypertension (35.58%), and dyslipidemia (21.28%). Median ages were higher for those with diabetes (65 vs. 51 years, *p* < 0.0001), coronary disease (64 vs. 51 years, *p* < 0.0001), arterial hypertension (65 vs. 43 years, *p* < 0.0001), and dyslipidemia (66 vs. 47 years, *p* < 0.0001). BMI (Body Mass Index) classifications showed 33.77% were overweight, 21.21% obese, and 15.07% morbidly obese. Smokers were younger than non-smokers (48 vs. 54 years, *p* < 0.0001). *Conclusions*: The findings highlight the significant prevalence of CVRFs among socioeconomically disadvantaged populations in Western Romania. Socioeconomically disadvantaged populations exhibit a significantly higher prevalence of cardiovascular risk factors such as diabetes, impaired glucose regulation, hypertension, and dyslipidemia compared to their before known status.

## 1. Introduction

Cardiovascular disease (CVD) remains a significant public health challenge worldwide, with disproportionate risks observed in certain populations due to various social determinants of health (SDOHs) [1,2]. These determinants, including socioeconomic status (SES), have a profound influence on health outcomes, particularly in the context of CVD. Globally, SES is a well-documented predictor of cardiovascular health, where lower SES is consistently associated with higher CVD risk and poorer outcomes [3,4].

Romania has the highest percentage of people at risk of poverty or social exclusion among EU (European Union) member states, with 34.5% of its population affected, according to Eurostat data. In 2021, 95.4 million people across the EU, representing 21.7% of the population, were at risk of poverty or social exclusion. This includes those facing severe material and social deprivation or living in households with very low work intensity. Romania’s figures are notably higher than the EU average, with other countries like Bulgaria (32%), Greece, and Spain (both 28%) also having significant portions of their populations at risk. In contrast, the lowest rates were observed in Czechia (11%), Slovenia (13%), and Finland (14%) [5].

Focusing on the local context, West Romania is one of the regions with lower rates of people at risk of poverty or social exclusion among Romania’s regions (29.5%) [6]. In the West Development Region of Romania, with a population of 2,014,732, approximately 29.5% are at risk of poverty or social deprivation. This translates to roughly 594,346 people facing such challenges in this region [7]. But this region still presents a particularly vulnerable population, where the intersection of low income, limited education, housing instability, and restricted access to healthcare creates a complex web of risks for CVD [8,9,10]. Social determinants of health (SDOHs) play a crucial role in shaping cardiovascular health and contributing to disparities in risk factors, outcomes, and clinical care for CVD. These determinants span six domains: economic stability, education, food, neighborhood and physical environment, healthcare system, and community and social context [11,12].

In West Romania, as well as in all of the country, factors such as income, education, housing stability, and access to healthcare intersect to create a complex web of risk [13,14,15,16]. Addressing these social determinants is crucial for improving cardiovascular health. Furthermore, disadvantaged populations often grapple with unhealthy behaviors that contribute to CVD. These include smoking, poor dietary choices, physical inactivity, and substance abuse [1,8,17].

Access to healthcare services is another pivotal factor that influences cardiovascular health outcomes in this region [2,4]. Financial constraints, lack of health insurance, and inadequate primary care facilities are significant barriers that hinder effective prevention and management of CVD. Community-based programs offer potential solutions to bridge these gaps, emphasizing the need for targeted interventions [17].

By focusing on this region, this study aims to investigate the prevalence and impact of cardiovascular risk factors (CVRFs) among socioeconomically disadvantaged populations in Western Romania. Specifically, it seeks to identify the association between SES and the presence of various CVRFs, such as diabetes, hypertension, dyslipidemia, and smoking, within this population. This study also aims to highlight the extent of undiagnosed conditions and the disparity in health outcomes due to limited access to healthcare and unhealthy lifestyle choices. Ultimately, this research study seeks to inform targeted public health interventions to reduce these disparities and improve cardiovascular health outcomes in socioeconomically disadvantaged communities.

## 2. Materials and Methods

### 2.1. General Information

This article presents the results from the project conducted by European Social Fund through the Operational Programme Human Capital (POCU) and by the Timisoara Local Action Group, with the main aim of improving the health and quality of life of vulnerable people. Project title: “Detection of cardiovascular problems and awareness of the population regarding medical problems”; SMIS code: 149707. The duration of this study was two years (November 2021–November 2023). 

This study was conducted in partnership with “Neoclinic Concept” Clinique, where all eligible patients included in this study were evaluated by two cardiologists with over 10 years of experience. Our target group for this investigation consisted of the residents of Freidorf (~10,000 habitants), a suburban region located in Timisoara, the major city in Western Romania.

### 2.2. Sample Size

This study’s sample size was carefully determined to ensure the representativeness of the socioeconomically disadvantaged population of the West Development Region of Romania, where 29.5% of the 2,014,732 regional population is at risk of poverty or social exclusion. All eligible subjects have been included from a population of around 10,000 inhabitants. The sample was chosen to capture the variability and prevalence of cardiovascular risk factors (CVRFs) and to achieve adequate statistical power for detecting significant differences in these factors, with a 95% confidence level and 80% power. Additionally, the sample size balanced the need for robust statistical analysis with practical and ethical considerations, including resource limitations and patient confidentiality.

### 2.3. Selection Criteria

The inclusion criteria were based on Eurostat’s EU-SILC (European Union Statistics on Income and Living Conditions) benchmarks [18]. The inclusion criteria were as follows:

(a) Severe material deprivation rate (SMD): population unable to afford at least four out of nine predefined material items essential for an adequate quality of life. These items include housing, heating, durable goods, and access to healthcare.
(b) At-risk-of-poverty rate: people who are at risk of falling below the poverty threshold (typically set at 60% of the national median equivalized disposable income). Eurostat provides information on at-risk-of-poverty rates for various demographic groups, including age, gender, and household composition.
(c) Low work intensity indicator: we analyzed data related to low work intensity, focusing on people aged 18–59 years living in households where adults (aged 18–59, excluding students aged 18–24) worked a working time equal to or less than 20% of their total combined work-time potential during the previous year [18].

### 2.4. Study Design and Analysis

This analysis employed a retrospective observational design, where data were gathered from the medical records of eligible individuals. The research gained ethical approval from the Ethics Committee of “Neoclinic Concept” Clinique (approval number 1/2024).

The analysis of subject records was conducted to gather demographic information, including age, gender, place of origin, weight, and height. Additionally, we collected information on familial history of diabetes mellitus and cardiovascular disease, as well as personal pathologies such as diabetes mellitus, cardiovascular disease, blood pressure, and dyslipidemia. All of this information was recorded based on their medical history documentation. Smoking status analysis, systolic and diastolic blood pressure measurement, glucose level, cholesterol level, triglycerides, and HbA1c level were obtained in the course of the cardiological examination. All investigations and measurements of medical test values were carried out in the same laboratory at “Neoclinic Concept” Clinique, using the same equipment settings. The anamnesis, data collection, and cardiology consultations were consistently performed by one of the two cardiologists with over 10 years of experience (A.D., S.S).

The cut-offs for all values were selected according to international guidelines. For systolic and diastolic blood pressure, we used the “2023 ESH Guidelines for the management of arterial hypertension” [19]. The cut-off for fasting glucose level was defined according to the American Diabetes Association [20]. The specific cut-off values for HDL, LDLc, and fasting triglyceride measurements were chosen according to the Adult Treatment Panel III (ATP III) Update [21,22].

### 2.5. Statistical Analysis

The statistical analysis was performed using MedCalc^®^ Statistical Software version 22.030, created by MedCalc^®^ Software Ltd. in Ostend, Belgium. The software can be viewed at the URL https://www.medcalc.org and was accessed on 15 June 2024.

The data related to identification, clinical, and paraclinical aspects were carefully documented in a secured computerized database using Microsoft^®^ Excel^®^ for Microsoft 365 MSO (Version 2406 Build 16.0.17726.20078) 64-bit, which was released on 11 June 2024.

This study included a summary of patient demographics, clinical characteristics, and laboratory data. The results were presented by first summarizing the patient demographics, clinical characteristics, and laboratory data. The distribution of data was assessed using the Shapiro–Wilk test to determine whether the data followed a normal distribution. Based on the results of this test, non-parametric statistical methods were primarily employed due to significant departures from normal distribution. Specifically, the Mann–Whitney test was used to compare non-parametric parameters, while the independent t-test was applied for parametric parameters. Differences between groups were considered statistically significant if the *p*-value was less than or equal to 0.05. The results were presented as median values with interquartile ranges (IQR) for non-parametric data and as means with standard deviations for parametric data. This approach allowed for a clear and accurate presentation of the data, highlighting significant differences where applicable.

## 3. Results

### 3.1. Demographic Features

Out of a total of 1433 subjects included, 498 were male (34.75%). The median age for entire lot was 52 with IQR between 38 and 64.50. The median age for male and female gender are 51 and 52, respectively. The Mann–Whitney test suggested that there was no significant difference in medians between the two samples (*p* = 0.17). All included subjects were from the same urban area, the Freidorf suburb. The conditions that were investigated include hereditary–collateral antecedents (HCAs), such as diabetes and cardiovascular diseases, as well as personal pathologic antecedents (PPAs) such as diabetes, coronary disease, blood pressure, and dyslipidemia. The prevalence of these conditions is presented in Table 1.

Table 2 presents the classification of parameter categories according to the subjects’ analyzed values. The patient data reveal a diverse range of BMI classifications. The median value of BMI is 28, with an interquartile range between 24.04 and 32. The highest value is 56 kg/m^2^, while the lowest one is 15.05 kg/m^2^. A small percentage of patients, 2.30%, fall into the underweight category with a BMI of less than 18.5. A significant proportion of the patients, 27.63%, have a normal weight, with BMIs ranging from 18.5 to 24.9. The largest group, comprising 33.77% of the patients, is classified as overweight, with BMIs between 25 and 29.9. Additionally, 21.21% of patients are categorized as obese, having BMIs from 30 to 34.9. Lastly, 15.07% of patients are considered morbidly obese, with BMIs exceeding 35. 

The median systolic blood pressure is 130, with an IQR between 117 and 145 mmHg. The median diastolic blood pressure is 85, with an IQR between 77 and 94 mmHg. 

The blood glucose levels range from 62 to 377 mg/dL, suggesting significant variability among patients. The median value is 105.50 mg/dL, with an IQR between 97 and 118 mg/dL.

The total cholesterol level ranges from 86 to 404 mg/dL. The median value is 195 mg/dL, with an IQR between 164 and 225 mg/dL. HDL levels range from 18 to 127 mg/dL, with a median of 52 mg/dL (42, 61). LDLc levels range from 15 to 258 mg/dL, with a median of 113 mg/dL (IQR 88, 140).

Fasting triglyceride levels vary from 24 to 2333 mg/dL. The median value is 111 mg/dL, with an IQR of 77–163 mg/dL.

HbA1c levels vary from 4.80 to 14.50%. The median percentage is 6.2%, with an IQR between 5.70 and 7.

### 3.2. Age Distribution and Its Association with Hereditary and Personal Pathologic Antecedents

**Figure 1:**
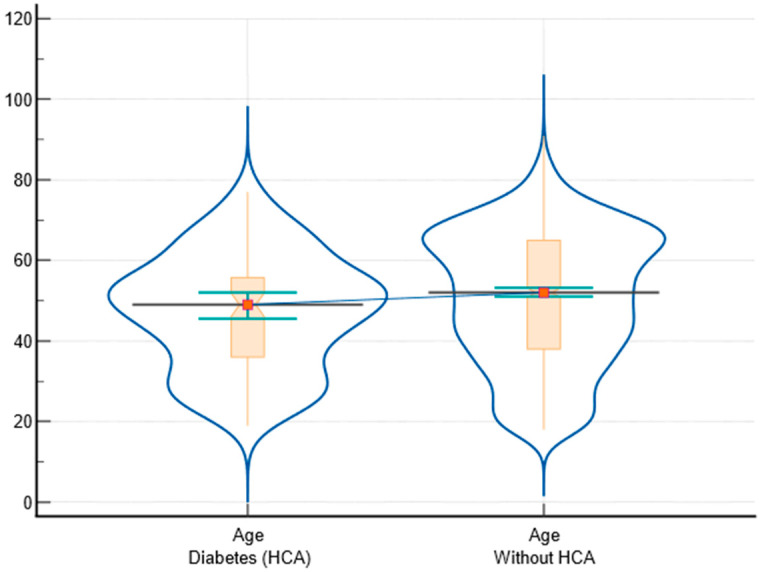
Graphical representation of age between subjects with and without HCA diabetes including notched box-and-whisker and violin plot representations (notched box-and-whisker, as well as horizontal lines, markers, connecting lines, and error bars, to indicate 95% confidence intervals for medians).

The ages of subjects with and without diabetes as an HCA have different median values (49 vs. 52), with a 95% confidence interval for the median difference between 1 and 7. The interquartile range also differs (36 to 55.75 vs. 38 to 65), indicating variability in the data. The Mann–Whitney test suggests a statistically significant difference between the groups (*p* = 0.014), with the second group having higher average ranks. The graphical representation is presented in Figure 1.

The ages of subjects with and without cardiovascular diseases as an HCA have different median values (48 vs. 52), with a 95% confidence interval for the median difference between 1 and 7. The interquartile range also differs (36 to 59 vs. 38.75 to 65), indicating variability in the data. The Mann–Whitney test suggests a statistically significant difference between the groups (*p* = 0.006), with the second group having higher average ranks. 

The median age of individuals diagnosed with diabetes was 65 years (57, 70), whereas the median age of individuals without diabetes was 51 years (37, 64). The disparity is extremely significant, as indicated by a *p*-value of less than 0.0001. 

The median age of individuals diagnosed with coronary disease was 64 years (57.50, 69.75), whereas those without the condition had a median age of 51 years (38, 64). The observed difference was extremely significant, as indicated by a *p*-value of less than 0.0001.

The median age of individuals diagnosed with blood pressure was 65 years (57, 70), whereas the median age of individuals without hypertension was 43 years (32, 55). The difference was extremely significant, as indicated by a *p*-value of less than 0.0001.

The median age of individuals diagnosed with dyslipidemia was 66 years (58, 70), while those without dyslipidemia had a median age of 47 years (34, 60). The observed difference was extremely significant, with a *p*-value of less than 0.0001.

The median age of smokers was 48 years (36, 62), while the median age of non-smokers was 54 years (40, 65). The difference was highly significant, with a *p*-value of less than 0.0001. Table 3 presents the differences between the ages of the subjects according to the investigated conditions.

### 3.3. Body Mass Index (BMI) Comparison across Hereditary and Personal Pathologic Conditions

Subjects with diabetes (both HCA and PPA) have a higher average BMI compared to those without diabetes, with significant differences. For cardiovascular conditions (HCA), there is no significant difference in BMI between subjects with and without the condition. Subjects with coronary disease (PPA) and blood pressure (PPA) also have higher average BMIs compared to those without these conditions, with significant differences. Similarly, subjects with dyslipidemia (PPA) have a higher average BMI compared to those without, with significant differences. Lastly, smokers have a slightly lower average BMI compared to non-smokers, with a significant difference. Table 4 presents the difference between BMI of subjects according to their condition status.

### 3.4. Gender-Based Variations in Cardiovascular and Metabolic Risk Factors

**Figure 2:**
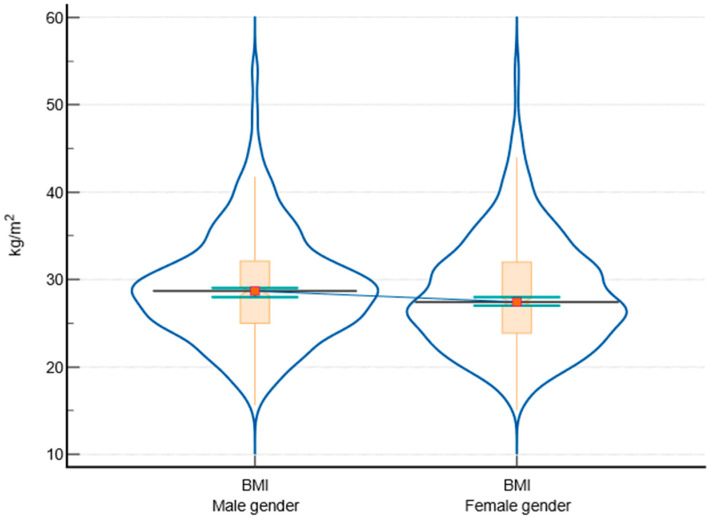
Graphical depiction of BMI between male and female participants, including notched box-and-whisker and violin plot representations (notched box-and-whisker, as well as horizontal lines, markers, connecting lines, and error bars, indicate 95% confidence intervals for medians).

**Figure 3:**
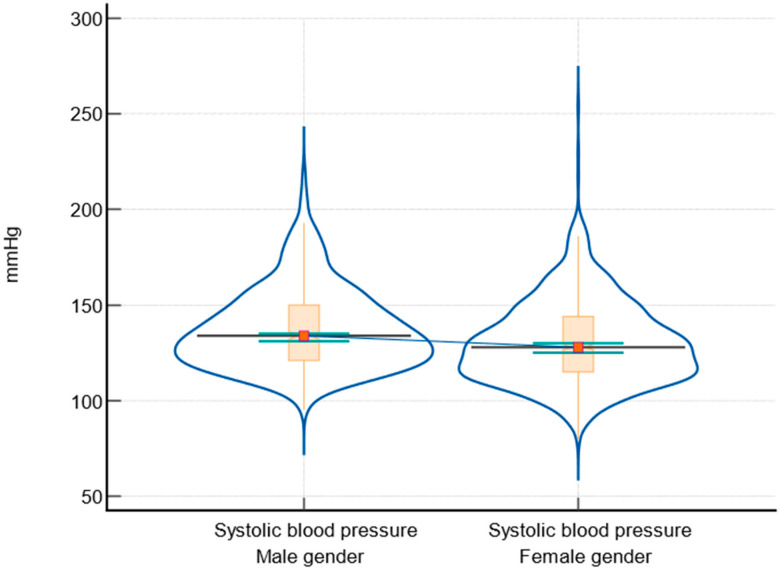
Graphical representation of systolic blood pressure between male and female participants, including notched box-and-whisker and violin plot representations (notched box-and-whisker, as well as horizontal lines, markers, connecting lines, and error bars, indicate 95% confidence intervals for medians).

**Figure 4:**
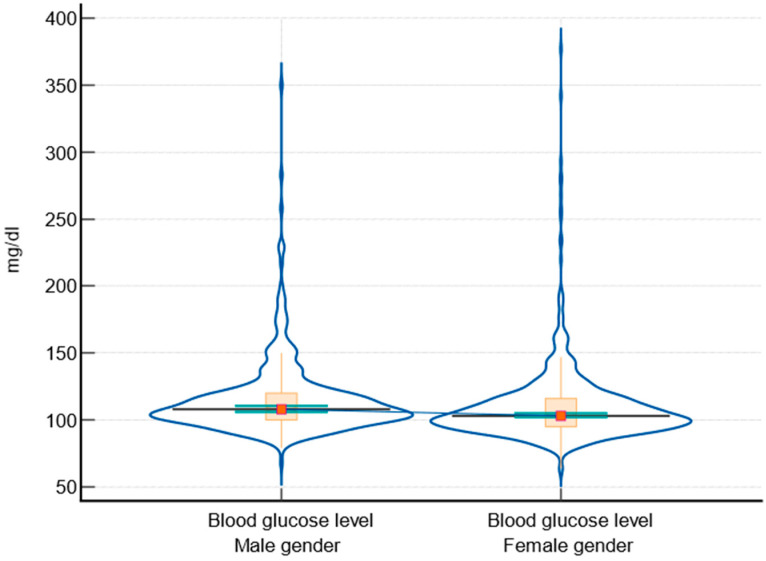
Graphical representation of blood glucose levels between male and female participants, including notched box-and-whisker and violin plot representations (notched box-and-whisker, as well as horizontal lines, markers, connecting lines, and error bars, indicate 95% confidence intervals for medians).

The data showed that males generally have higher BMI (Figure 2), systolic (Figure 3) and diastolic blood pressure, blood glucose levels (Figure 4), and fasting triglyceride levels compared to females. However, females tended to have higher HDL levels. There were no significant differences in total cholesterol and LDLc levels between males and females. HbA1c levels were slightly higher in males, but the difference was not statistically significant. The comparison between male and female participants according to parameter variabilities is presented in Table 5.

### 3.5. Prevalence and Distribution of Cardiovascular Risk Factors in the Study Population

Approximately 25% of the included subjects (345 patients) did not have a prior history of hypertension (no previous physician-diagnosed hypertension). Despite this, their blood pressure measurements indicated they could be classified as having at least grade I hypertension. Additionally, a substantial 41.59% (596 patients) of those without a history of dyslipidemia (no previous physician-diagnosed dyslipidemia) had LDLc levels higher than 100 mg/dL. 

**Figure 5:**
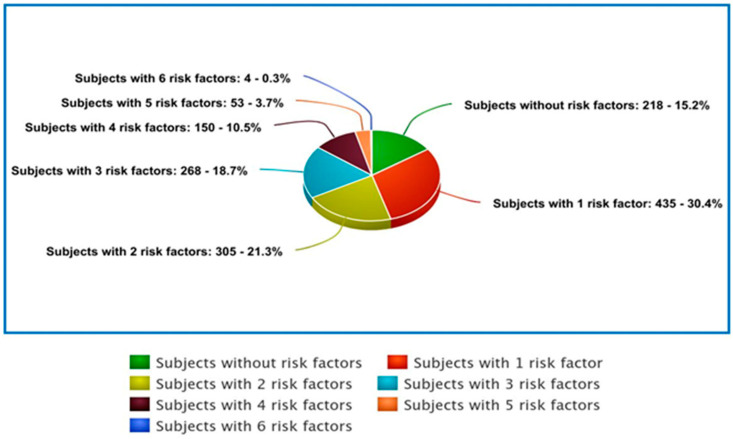
Pie chart graphical representation of included subjects.

Table 6 displays the classification of the included patients according to the risk factors (BMI > 30 kg/m^2^, smoker, SBP ≥ 140 mmHg, DBP ≥ 90 mmHg, fasting glucose ≥ 126 mg/dL, and LDLc ≥ 130 mg/dL) they are related to. Figure 5 presents the distribution of these categories.

### 3.6. Relative Risk (RR) and Odds Ratio (OR) for Various Outcomes Based on Associated Risk Factors

Individuals with a family history of diabetes (HCA of diabetes) are at a much higher risk of developing diabetes themselves. A higher body mass index (BMI) is strongly linked to elevated blood glucose levels, with the risk increasing as BMI rises. Elevated systolic and diastolic blood pressure levels are also significantly associated with higher blood glucose levels, indicating a strong interplay between hypertension and glucose regulation. Although the presence of a family history of cardiovascular disease (HCA of cardiovascular conditions) slightly increases the risk of coronary disease, this association was not statistically significant. The associations between CVRFs and disease outcomes are presented in Table 7.

## 4. Discussion

### 4.1. Cardiovascular Risk Factors among Socioeconomically Disadvantaged Populations in Western Romania: A Comparative Analysis with National and International Studies

This study highlighted a significant prevalence of cardiovascular risk factors among the socioeconomically disadvantaged population in West Romania. PPA conditions such as arterial hypertension (35.58%), dyslipidemia (21.28%), and diabetes (7.39%) were notably prevalent. These findings are consistent with numerous studies that emphasize the disproportionate burden of CVRFs in low-socioeconomic-status populations. For example, a study conducted by Barnett et al. found that individuals in lower SES brackets have a higher prevalence of risk factors such as hypertension, diabetes, and smoking compared to higher SES groups. This is attributed to limited access to healthcare, unhealthy lifestyle choices, and greater psychosocial stress [23]. Furthermore, Kanjilal et al. highlighted that low SES is strongly associated with higher incidences of hypertension and dyslipidemia, paralleling the findings in this West Romania study [24].

The analysis showed significant differences in the median ages of subjects with and without CVRFs. For example, individuals with diabetes had a median age of 65 years, compared to 51 years for those without diabetes. Similarly, those with coronary disease and arterial hypertension also had higher median ages. These findings are similar to others that demonstrated that SES-related disparities significantly impact the onset and progression of CVD. Higher-SES individuals tend to have better access to preventive measures and treatment options, delaying the onset of CVD. Disadvantaged neighborhoods exhibited higher CVD prevalence and poorer health outcomes, emphasizing the role of social determinants in health [25,26,27].

Our data showed a distribution of BMI classifications among a group of individuals, with the majority falling into the overweight category (33.77%), followed by normal weight (27.63%), obese (21.21%), morbidly obese (15.07%), and underweight (2.30%). This distribution highlights the prevalence of higher BMI categories within the group, which is a common trend observed in various populations globally. Comparing these results with similar studies, a study using the UK Biobank cohort identified similar trends, with a significant portion of the population classified as overweight or obese. This study also highlighted the complexity of obesity, considering various factors like societal influences, individual psychology, and physical activity. Such findings underscore the multifaceted nature of obesity and the need for comprehensive strategies to address it [28,29]. Furthermore, they highlight the ongoing global challenge of obesity and the need for comprehensive approaches to address it. Understanding these trends and their implications can help in developing targeted interventions to improve health outcomes [30,31].

For the HCA of diabetes, the BMI of subjects with diabetes (29.62) was significantly higher than that of those without (27.74), with a *p*-value of 0.011. This finding aligns with the existing literature, which suggests higher BMI is a risk factor for developing diabetes, particularly in individuals with a family history of the condition [32]. 

In the case of the cardiovascular HCA, the difference in BMI between subjects with cardiovascular conditions (28.30) and those without (28) was not statistically significant (*p*-value 0.847). This suggests that while BMI is an important factor, other hereditary factors might play a more critical role in cardiovascular health [33,34]. This suggests that while BMI is a factor, other elements such as genetic predisposition, lifestyle, and other comorbidities play a crucial role in cardiovascular health. Similar findings were reported in a study in the *Journal of the American College of Cardiology*, which emphasized the multifactorial nature of cardiovascular disease [1,35]. 

Regarding the PPAs of diabetes, coronary disease, arterial hypertension, and dyslipidemia, the BMI of subjects with these conditions was significantly higher than that of those without. This strong association underscores the impact of personal health history on these diseases’ risk, consistent with the finding that higher BMI is a significant risk factor for diabetes, coronary disease, arterial hypertension, and dyslipidemia [36,37].

The active smoking rate of 31.71% is notably high. This is consistent with other research indicating a higher smoking prevalence in socioeconomically disadvantaged populations compared to the general population. Nationally, the smoking rate tends to be lower, often around 14–15% in many developed countries [38]. Furthermore, the percentage of ex-smokers (9.79%) is relatively low. This suggests potential barriers to quitting smoking within this population, such as limited access to smoking cessation programs, lack of healthcare support, and higher levels of stress and mental health issues, which are known to affect smoking cessation efforts. Studies consistently show higher smoking rates among socioeconomically disadvantaged groups. For example, research published in the USA indicated that smoking prevalence in the lowest income brackets can be as high as 30–35%, aligning with the 31.71% observed in our data [39,40].

Regarding smoker status, the BMI of smokers (27.66) was slightly lower than that of non-smokers (28), with a *p*-value of 0.031. This finding is intriguing and aligns with the hypothesis that smoking may suppress appetite and increase metabolism, leading to lower body weight. Furthermore, this small but significant difference is consistent with findings in the *American Journal of Public Health*, which suggest that smokers tend to have a lower BMI but higher risks of other health issues [41,42].

Regarding the BMI of the subjects, the analyzed data highlighted significant differences in cardiovascular risk factors between males and females. Firstly, males have a higher median BMI compared to females, with a statistically significant difference (*p* = 0.021). This aligns with studies showing that males in disadvantaged populations often have higher BMI due to lifestyle factors and limited access to healthy food options [3]. Both systolic and diastolic blood pressures were higher in males, with highly significant *p*-values (<0.0001 and 0.001, respectively). These findings are similar to those of the SEPHAR study, which included people from the same geographic area [43,44]. High blood pressure is more prevalent in socioeconomically disadvantaged areas, often due to stress, poor diet, and lack of healthcare access, contributing to increased cardiovascular risk [2,36,45]. Also, males had higher blood glucose levels than females, with a significant *p*-value (<0.0001). This is consistent with findings that disadvantaged populations have higher rates of diabetes and poor glycemic control due to limited healthcare access [17,46]. 

It is important to note that the occurrence of diabetes, based on their medical records, was 7.39%. However, 16.41% of individuals had a fasting glucose level exceeding 126 mg/dL. In contrast, the PREDATORR study, conducted from 2012 to 2014, found a diabetes prevalence of 11.6% among the adult Romanian population [46]. A deep analysis of these data showed that impaired glucose regulation (prediabetes, known and unknown diabetes) was found in 28.1% of the study population in the PREDATORR study, compared to 68.11% of our study population with fasting glucose levels higher than 100 mg/dL [46]. The early diagnosis of diabetes is crucial in preventing or delaying the progression of the disease and the onset of complications, such as cardiovascular diseases, neuropathy, and nephropathy, which significantly impact patient outcomes and healthcare resources [47,48,49].

Total cholesterol levels were slightly higher in females, but the difference was not statistically significant (*p* = 0.062). HDL levels were significantly higher in females (<0.0001), while LDL levels showed no significant difference (*p* = 0.562). Disadvantaged populations often have poor lipid profiles due to dietary habits and lack of preventive care [11]. However, regardless of fasting triglyceride levels, males had increased levels. Elevated triglycerides are common in socioeconomically disadvantaged groups, linked to diet and metabolic syndrome [11,50]. 

The results of this study align with existing research on cardiovascular risk factors in socioeconomically disadvantaged populations, highlighting significant associations between these risk factors and health outcomes. The strong relationship between diabetes and a family history of the disease (HCA) underscores the genetic predisposition in these populations, as supported by studies like those by Sirdah et al. and Wang et al. [51,52]. Furthermore, the significant increase in the risk for elevated blood glucose levels in individuals with higher BMI (≥25 kg/m^2^ and ≥30 kg/m^2^) is consistent with findings from Wondmkun’s research, which noted a strong link between obesity and impaired glucose regulation in low-income populations [53]. The progressive increase in the risk of hypertension with elevated blood glucose levels also mirrors the Framingham Heart Study’s findings, which highlighted the connection between hyperglycemia and hypertension in at-risk populations [54,55]. Additionally, the observed, albeit not statistically significant, association between coronary disease and a family history of cardiovascular disease suggests a hereditary component that may be influenced by lifestyle and environmental factors, as noted in the INTERHEART study [56,57]. Overall, these findings emphasize the compounded cardiovascular risks faced by disadvantaged populations, reinforcing the need for targeted public health interventions that address both genetic predispositions and socioeconomic factors.

### 4.2. Comparative Analysis of Cardiovascular Risk Factors in Socioeconomically Disadvantaged Populations: Insights from Recent Romanian Studies

Our study’s findings align with and expand upon those in the attached article by Simionescu et al., which examined CVD risk factors and mortality in Romania. Both studies highlight the significant burden of CVDs, particularly among socioeconomically disadvantaged populations, with our research identifying a high prevalence of arterial hypertension, dyslipidemia, and diabetes, which mirrors Simionescu et al.’s emphasis on hypertension as a leading risk factor. While Simionescu et al. discuss the persistent high mortality rates despite increased healthcare infrastructure, our study adds depth by examining the significant correlations between BMI, smoking, and cardiovascular risk factors, reinforcing the complex interplay of lifestyle factors and CVD risk. Both studies underscore the need for enhanced healthcare access and targeted public health policies to address these risk factors, particularly in disadvantaged communities, presenting a compelling case for prioritizing cardiovascular health in Romania’s public health strategy [9].

The findings of our study, which highlighted a significant prevalence of CVRFs such as arterial hypertension, dyslipidemia, and diabetes among a socioeconomically disadvantaged population in West Romania, align closely with the results of several key studies in Romania, including those by Simionescu et al., Rosu et al., Weiss et al., and Cinteza et al. (CARDIO-Zone study) [9,58,59,60]. Both our study and the CARDIO-Zone study reveal high rates of hypertension and diabetes, although our study found even higher rates of undiagnosed cases, particularly of hypertension and dyslipidemia, suggesting gaps in healthcare access and preventive care. Additionally, while the CARDIO-Zone study emphasized regional differences in CVRF prevalence, our research adds to this by examining the complex interplay of BMI, smoking, and CVRFs, further reinforcing the role of lifestyle and socioeconomic factors in cardiovascular health [60]. Moreover, the prevalence of obesity and smoking in our study mirrors that observed in broader Romanian studies, indicating consistent patterns across different populations and regions. Overall, these findings collectively underscore the urgent need for targeted public health interventions and improved healthcare accessibility to mitigate the burden of cardiovascular diseases in Romania, particularly among disadvantaged communities.

All of these findings align with broader research indicating that socioeconomically disadvantaged populations face higher cardiovascular risks due to a combination of biological, behavioral, and psychosocial factors [4,61]. Furthermore, the data from the current study and the PREDATORR and SEPHAR studies collectively indicate high rates of hypertension and metabolic disorders among the Romanian population, particularly in socioeconomically disadvantaged groups. The high prevalence of active smokers in the current study (31.71%) exacerbates these health conditions, highlighting the need for targeted interventions [8,43,44,46].

### 4.3. Strategies to Reduce Cardiovascular Risk in Socioeconomically Disadvantaged Populations: Addressing Health Equity through Targeted Interventions and Policy Initiatives within the Romanian Population

Efforts to achieve health equity should address the structural, institutional, and environmental barriers faced by marginalized communities. By considering SDOHs, we can inform CVD care, ensure equitable healthcare resource distribution, and reduce observed disparities [3]. To reduce the high prevalence of CVRFs in socioeconomically disadvantaged populations, several targeted interventions could be implemented, focusing on both systemic and community-level strategies [9]. First, increasing access to healthcare is crucial, particularly in underserved areas, through the expansion of community health centers and mobile clinics. These facilities can provide essential services such as regular health screenings, early diagnosis, and the management of conditions like hypertension, diabetes, and dyslipidemia, which are often undiagnosed in these populations. Second, public health campaigns tailored to the specific needs and circumstances of disadvantaged communities could raise awareness about the importance of healthy lifestyles, including diet, physical activity, and smoking cessation. These campaigns should be culturally sensitive and involve community leaders to enhance their effectiveness. Third, implementing policies that address social determinants of health, such as improving access to affordable healthy food options and creating safe spaces for physical activity, is essential. For example, incentivizing grocery stores to stock fresh produce in food deserts or developing urban green spaces can significantly impact community health. Fourth, integrating mental health support into cardiovascular care is important, as chronic stress related to socioeconomic disadvantage contributes to the risk of CVD. Providing mental health services within primary care settings could help mitigate these effects. Lastly, policy-level interventions, such as implementing taxes on sugary drinks and tobacco products, alongside subsidies for healthy food options, could further encourage healthier behaviors. Collectively, these strategies could substantially reduce the burden of CVRFs in socioeconomically disadvantaged populations, leading to improved health outcomes and reduced healthcare disparities [9,62,63,64,65].

### 4.4. Limitations

This study employed a retrospective observational design, which inherently limits the ability to establish causal relationships between socioeconomic factors and CVD outcomes. Observational studies can show associations but not causation, and the findings may be influenced by unmeasured confounding variables. Moreover, factors such as diet, physical activity, alcohol consumption and lifestyle behaviors were not taken into account.

This study was conducted within a specific geographical area (West Romania) and may not be representative of other regions or populations. Furthermore, some analyses within this study were cross-sectional, examining data at a single point in time. This approach does not account for changes in socioeconomic status or health behaviors over time, which can significantly influence cardiovascular health outcomes.

### 4.5. Future Directions

Future directions should include the integration of digital health technologies and personalized medicine into socioeconomically disadvantaged populations. For example, the use of mobile health (mHealth) applications and telemedicine can play a crucial role in improving access to care in underserved areas. These technologies can facilitate the remote monitoring of cardiovascular risk factors such as blood pressure and glucose levels, enabling timely interventions and continuous patient engagement [66,67]. Additionally, implementing community-based participatory research can empower these populations by involving them in the design and implementation of health interventions, ensuring that the strategies are culturally relevant and more likely to be effective [68]. Another innovative approach could involve the use of genetic screening and precision medicine to identify individuals at higher risk due to hereditary factors, allowing for more targeted and personalized prevention and treatment plans [52,53]. These strategies collectively represent a more holistic and tailored approach to reducing the burden of cardiovascular risk factors in socioeconomically disadvantaged populations [1,30,31].

## 5. Conclusions

In conclusion, socioeconomically disadvantaged populations exhibit a significantly higher prevalence of cardiovascular risk factors such as diabetes, impaired glucose regulation, hypertension, and dyslipidemia compared to their previously known status. These disparities are likely due to limited access to healthcare, unhealthy lifestyle choices, and greater psychosocial stress, which compound the risk of developing chronic conditions. Therefore, enhanced attention and targeted public health interventions are essential to address these health inequities. Prioritizing preventive care, early diagnosis, and accessible treatment options for these communities is crucial in reducing the burden of cardiovascular diseases and improving overall health outcomes.

## Figures and Tables

**Table 1:** The prevalence of the investigated conditions.

Condition	Number of Subjects	Percentage
HCA (diabetes)	115	8.02%
HCA (cardiovascular)	126	8.79%
PPA (diabetes)	106	7.39%
PPA (coronary disease)	55	3.83%
PPA (blood pressure)	510	35.58%
PPA (dyslipidemia)	305	21.28%

HCA—hereditary–collateral antecedent; PPA—personal pathologic antecedent.

**Table 2:** Classification of parameter categories according to subjects’ analyzed values.

Category	Parameter	No of Subjects (Percentage)
	**BMI**	
Underweight	<18.5 kg/m^2^	33 (2.30%)
Normal weight	18.5–24.9 kg/m^2^	396 (27.63%)
Overweight	25–29.9 kg/m^2^	484 (33.77%)
Obese	30–34.9 kg/m^2^	304 (21.21%)
Morbidly obese	≥35 kg/m^2^	216 (15.07%)
	**Smoker status**	
Non-smoker		830 (58.49%)
Active smoker		450 (31.71%)
Ex-smoker	Smoke-free for at least 28 days	139 (9.79%)
	**Systolic blood pressure**	
Optimal	<120 mmHg	394 (27.84%)
Normal	120–129 mmHg	282 (19.92%)
High normal	130–139 mmHg	251 (17.73%)
Grade 1 hypertension	140–159 mmHg	319 (22.54%)
Grade 2 hypertension	160–179 mmHg	117 (8.26%)
Grade 3 hypertension	≥180 mmHg	52 (3.67%)
	**Diastolic blood pressure**	
Optimal	<80 mmHg	400 (28.24%)
Normal	80–84 mmHg	263 (18.57%)
High normal	85–89 mmHg	220 (15.53%)
Grade 1 hypertension	90–99 mmHg	315 (22.24%)
Grade 2 hypertension	100–109 mmHg	155 (10.94%)
Grade 3 hypertension	≥110 mmHg	63 (4.68%)
	**Fasting blood glucose level**	
Normal	<100 mg/dL	381 (31.90%)
Alteration in fasting glucose	100–125 mg/dL	617 (51.67%)
Alteration in fasting glucose (diabetes category)	≥126 mg/dL	196 (16.41%)
	**HDL level**	
Low	<40 mg/dL	218 (18.07%)
Intermediate	40–59 mg/dL	637 (52.81%)
High	≥60 mg/dL	351 (29.10%)
	**LDLc level**	
Optimal	< 100 mg/ dL	451 (37.83%)
Near optimal/above optimal	100–129 mg/dL	331 (27.76%)
Borderline high	130–159 mg/dL	238 (19.96%)
High	160–189 mg/dL	127 (10.65%)
Very high	≥190 mg/dL	45 (3.77%)
	**Fasting triglyceride level**	
Normal	<150 mg/dL	840 (69.65%)
Mild hypertriglyceridemia	150–499 mg/dL	352 (29.18%)
Moderate hypertriglyceridemia	500–886 mg/dL	11 (0.91%)
Very high or severe hypertriglyceridemia	>886 mg/dL	3 (0.24%)

BMI—body mass index.

**Table 3:** Age of subjects according to investigated conditions.

Condition	Age of Subjects with Condition	Age of Subjects without Condition	*p*-Value
HCA (diabetes)	49; (36, 55.75)	52; (38, 65)	0.014
HCA (cardiovascular)	48; (36, 59)	52; (38.75, 65)	0.006
PPA (diabetes)	65; (57, 70)	51; (37, 64)	<0.001
PPA (coronary disease)	64; (57.50, 69.75)	51; (38, 64)	<0.001
PPA (blood pressure)	65; (57, 70)	43; (32, 55)	<0.001
PPA (dyslipidemia)	66; (58, 70)	47; (34, 60)	<0.001
Smoker status	48; (36, 62)	54; (40, 65)	<0.001

HCA—hereditary–collateral antecedent; PPA—personal pathologic antecedent. Data are presented as medians (IQRs).

**Table 4:** BMI of subjects according to condition status.

Condition	BMI of Subjects with Condition	BMI of Subjects without Condition	*p*-Value
HCA (diabetes)	29.62; (25.77, 34.56)	27.74; (24, 32)	0.011
HCA (cardiovascular)	28.30; (23.70, 32.87)	28; (24.15, 32)	0.847
PPA (diabetes)	31.63; (28.53, 36.07)	27.64; (24, 31.80)	<0.001
PPA (coronary disease)	29.36; (25.95, 34.58)	27.93; (24.02, 32)	0.033
PPA (blood pressure)	30; (27, 34.44)	26.53; (23, 31.04)	<0.001
PPA (dyslipidemia)	29.40; (26.37, 34)	27.10; (23.70, 31.96)	<0.001
Smoker status	27.66; (23.90; 31.71)	28; (24.52, 32.36)	0.031

HCA—hereditary–collateral antecedent; PPA—personal pathologic antecedent.

**Table 5:** Comparison between male and female participants according to parameter variabilities.

Parameter	Male Gender	Female Gender	*p*-Value
BMI	28.70; (25, 32.11)	27.43; (23.87, 32)	0.021
Systolic blood pressure	134; (121, 150)	128; (115,144)	<0.001
Diastolic blood pressure	86; (80, 96)	85; (76, 92)	0.001
Blood glucose level	108; (100, 120)	103; (95, 116)	<0.001
Total cholesterol level	191; (160.75, 221)	196; (166, 228)	0.062
HDL level	45; (38, 55)	54; (45, 64)	<0.001
LDLc level	111; (87, 143)	114; (88, 140)	0.562
Fasting triglyceride level	125; (86, 191.25)	107; (74, 153.25)	<0.001
HbA_1c_	6.2; (5.80, 7.45)	6.1; (5.70, 6.87)	0.487

**Table 6:** The classification of the included subjects, according to the risk factors they are related to.

Classification	Number of Subjects	Percentage
Subjects without risk factors	218	15.21%
Subjects with 1 risk factor	435	30.35%
Subjects with 2 risk factors	305	21.28%
Subjects with 3 risk factors	268	18.70%
Subjects with 4 risk factors	150	10.46%
Subjects with 5 risk factors	53	3.69%
Subjects with 6 risk factors	4	0.27%

**Table 7:** Associations between cardiovascular risk factors and disease outcomes: relative risk and odds ratios.

Outcome	Group	RR (*p*-Value)	OR (*p*-Value)
Diabetes	HCA (diabetes)	3.5 (<0.001)	4.19 (<0.001)
Blood glucose level ≥ 126 mg/dL	BMI ≥ 25 kg/m^2^	3.2 (<0.001)	3.76 (<0.001)
Blood glucose level ≥ 126 mg/dL	BMI ≥ 30 kg/m^2^	2.26 (<0.001)	2.68 (<0.001)
SBP ≥ 140 mmHg	Blood glucose level ≥ 126 mg/dL	1.66 (<0.001)	2.48 (<0.001)
SBP ≥ 160 mmHg	Blood glucose level ≥ 126 mg/dL	2.56 (<0.001)	3.12 (<0.001)
SBP ≥ 180 mmHg	Blood glucose level ≥ 126 mg/dL	2.54 (<0.001)	2.68 (0.001)
DBP ≥ 90 mmHg	Blood glucose level ≥ 126 mg/dL	1.37 (<0.001)	1.81 (<0.001)
DBP ≥ 100 mmHg	Blood glucose level ≥ 126 mg/dL	1.61 (<0.001)	1.82 (0.001)
DBP ≥ 110 mmHg	Blood glucose level ≥ 126 mg/dL	1.69 (0.066)	1.75 (0.068)
PPA (coronary disease)	HCA (Cardiovascular)	1.71 (0.144)	1.76 (0.148)

## Data Availability

The information is contained within this article in its entirety. For additional information, please feel free to inquire with either the original author or the corresponding author. Public access to the data is restricted as a result of the patient privacy standards that regulate the handling of clinical data.

## Abbreviations

CVD	cardiovascular disease
SDOHs	social determinants of health
SES	socioeconomic status
CVRFs	cardiovascular risk factors
EU	European Union
EU-SILC	European Union Statistics on Income and Living Conditions
SMD	severe material deprivation rate
IQR	interquartile range
HCA	hereditary–collateral antecedents
PPA	personal pathologic antecedents
BMI	body mass index
SBP	systolic blood pressure
DBP	diastolic blood pressure
HDL	High-Density Lipoprotein
LDLc	Low-Density Lipoprotein Cholesterol
ATP III	Adult Treatment Panel III
HbA1c	Hemoglobin A1c
